# CTGF is a central mediator of tissue remodeling and fibrosis and its inhibition can reverse the process of fibrosis

**DOI:** 10.1186/1755-1536-5-S1-S24

**Published:** 2012-06-06

**Authors:** Kenneth E Lipson, Carol Wong, Yuchin Teng, Suzanne Spong

**Affiliations:** 1 FibroGen, Inc., 409 Illinois St., San Francisco, CA 94158, USA

## Abstract

CTGF is a secreted matricellular protein with very complex biology. It has been shown to modulate many signaling pathways leading to cell adhesion and migration, angiogenesis, myofibroblast activation, and extracellular matrix deposition and remodeling, which together lead to tissue remodeling and fibrosis. It has been reported in the literature that inhibition of CTGF expression by siRNA prevents CCl_4_-induced liver fibrosis and can reverse fibrosis when administered after significant collagen deposition is observed. A monoclonal antibody to CTGF that is currently in clinical development (FG-3019) has demonstrated the ability to reverse vascular stiffening and improve cardiac function in a rat model of diabetic complications. FG-3019 has also exhibited activity in a murine radiation-induced pulmonary fibrosis model. When FG-3019 was administered to mice after a significant radiation-induced increase in lung density could be observed by CT imaging, the density of the lungs was observed to decrease over the period during which the antibody was administered and to remain stable after therapy had ceased. When considered together, these data indicate that inhibition of CTGF can prevent and reverse the process of fibrosis.

## Introduction

### CTGF and its modulation of cell biology

Connective tissue growth factor (CTGF, CCN2) is a member of a small family of proteins that are characterized by their highly conserved disulfide bonding pattern and having 3-4 domains with homology to other proteins [1,2]. CTGF has four domains: domain 1 is homologous to IGF-1 binding proteins, domain 2 is homologous to the von Willebrand factor type C repeat, domain 3 is homologous to the thrombospondin type 1 repeat and domain 4 contains a cysteine knot motif that is common to proteins that bind to heparan sulfate proteoglycans (HSPGs). The two N-terminal domains (1 and 2) are linked to the two C-terminal domains (3 and 4) by a peptide that is not homologous to other proteins and is proteolytically labile. The N-half of CTGF is proteolytically stable and can often be observed in biological fluids (plasma, urine) of diseased subjects, but is rarely observed at substantial levels in healthy individuals [3,4]. The C-half of CTGF appears to be proteolytically labile, and is rarely observed in biological fluids. Similarly whole CTGF is rarely observed at significant concentrations in biological fluids, except in subjects with liver disease [5,6].

Although it was called CTGF when it was discovered [7], it does not behave like a traditional growth factor or cytokine since it does not appear to have a unique receptor to which it binds with high affinity to induce signal transduction. It may be more accurate to think of CTGF as a matricellular protein that modulates the interaction of cells with the matrix to modify the cellular phenotype [8-11]. Matricellular proteins such as SPARC, osteopontin and thrombospondins represent a sub-class of extracellular matrix (ECM) proteins that do not provide a structural function, but instead modulate cellular functions and signaling through multiple mechanisms that depend on the cell type and context [8].

**Figure 1:**
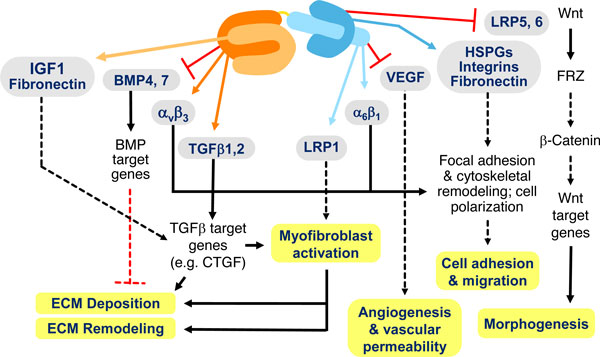
**CTGF affects multiple signaling pathways and processes important in pathophysiology**. CTGF interacts with a variety of molecules, including cytokines and growth factors, receptors and matrix proteins. These interactions alter signal transduction pathways, either positively or negatively, which results in changes in cellular responses.

CTGF has been reported to interact with a variety of molecules after being secreted from cells (Figure 1). Some of the molecules with which CTGF has been reported to interact are cytokines and growth factors such as IGF1, BMP4, BMP7, TGFβ, and VEGF. Other molecules are cell surface receptors with other known ligands such as TrkA, LRP1, LRP6 and several different integrins. CTGF has also been reported to interact with matrix proteins such as fibronectin or heparan sulfate proteoglycans (HSPGs) that may be in the matrix or on the cell surface. Interaction with each of these various molecules has been reported to be dependent on the different domains of CTGF.

The interaction of CTGF with various molecules is thought to positively or negatively alter the signal transduction pathways in which they participate. The result of this modulation of signaling are changes in cell adhesion and migration, angiogenesis and vascular permeability, differentiation, including myofibroblast formation and activation, extracellular matrix deposition and remodeling, all of which together lead to tissue remodeling and changes in organ structure.

Although the dogma in the CTGF literature suggests that all of the biological responses to CTGF are mediated by the direct interaction of CTGF with cytokines or receptors, the mechanisms by which it modulates cell function are likely to be more complicated. There are at least 5 mechanisms by which CTGF could simultaneously modulate the cellular environment and phenotype: 1) CTGF could act as an extracellular adapter protein by binding to cytokines and helping to present them to their receptors to stimulate response (e.g. TGFβ [12]) or sequestering them in the matrix and thereby preventing them from stimulating signal transduction (e.g. VEGF [13]); 2) CTGF could competitively bind to HSPGs, which would displace heparin-binding growth factors or receptor antagonists and thereby alter their local concentration and ability to modulate signaling (data not shown); 3) CTGF can block matrix binding sites or create new matrix binding sites, which would alter matrix signaling, cell adhesion and motility (e.g. [14-16]); 4) CTGF can bind directly to cell surface receptors and stimulate signal transduction (e.g. [16-18]); and 5) CTGF may be taken up into cells via endocytic pathways and act as an intracellular adapter protein to modulate signal transduction pathways in the cytoplasm and/or nucleus [19]. Because CTGF can work through so many mechanisms simultaneously, its biology is very complicated and incompletely understood. In addition, response to CTGF will be context dependent, and will vary with matrix, cytokine environment and cell genotype. As a result, the literature is filled with apparent contradictions of the effects of CTGF.

### CTGF is a central mediator of tissue remodeling and fibrosis

**Figure 2:**
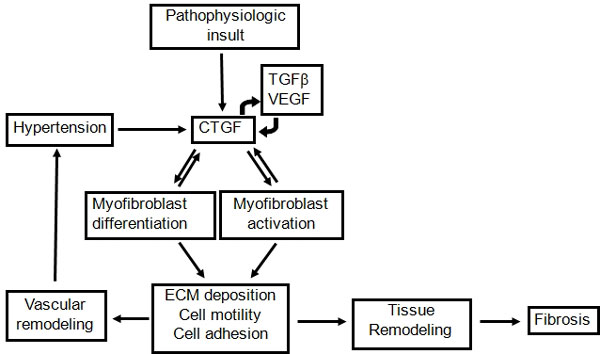
**CTGF is a central mediator of tissue remodeling and fibrosis**. Many different stimuli can induce expression of CTGF, which then promotes formation of myofibroblasts by modulating differentiation of other cells, including epithelial cells (EMT, epithelial to mesenchymal transition), resident fibroblasts or recruited fibrocytes (bone-marrow-derived, circulating mesenchymal stem cells). CTGF also promotes activation of the myofibroblasts and stimulates extracellular matrix (ECM) deposition and tissue remodeling. Remodeling in the vasculature can produce local hypertension that induces the expression of more CTGF, resulting in a positive feedback loop. Other positive feedback loops result from cytokines whose expression may be stimulated by CTGF, that in turn induce the expression of CTGF.

Regardless of the complexity of the mechanism of action by which CTGF modulates cell biology, it is very clear that CTGF plays a central role in diseases in which tissue remodeling occurs (Figure 2). CTGF expression is induced by many cytokines and conditions associated with pathophysiology [20]. Its presence induces formation of myofibroblasts through transdifferentiation of other cells, including epithelial cells (via EMT, epithelial to mesenchymal transition) [21], stellate cells [22], resident fibroblasts [23] or fibrocytes (bone-marrow-derived, circulating mesenchymal stem cells) that have been recruited to an organ through chemokines [24]. CTGF also activates the myofibroblasts and stimulates their deposition and remodeling of ECM (extracellular matrix) proteins. This leads to tissue remodeling and fibrosis. When the tissue remodeling occurs in the vasculature, it can create local hypertension that can induce CTGF expression [25-27], thereby setting up a positive feedback loop leading to more tissue remodeling. CTGF also induces the expression of a variety of cytokines such as TGFβ [28] and VEGF [29], which induce more expression of CTGF. Thus, there are multiple positive feedback loops involving CTGF expression that can contribute to the progressive nature of fibrosis. By inhibiting CTGF, these positive feedback loops can be broken, which should enable organs to restore their normal wound healing response and their normal structure and function.

CTGF has also been reported to inhibit BMP-7 signaling in diabetic kidney disease [30]. Since BMP-7 is thought to counteract the pro-fibrotic effects of TGFβ [31,32], inhibition of CTGF might also restore an anti-fibrotic regulatory pathway. Thus, inhibition of CTGF has the potential to modulate both pro- and anti-fibrotic mechanisms in a direction that could enable reversal of fibrotic processes.

### FG-3019

In order to identify an inhibitor of CTGF with the potential to be developed into a therapeutic agent, FibroGen screened a library of human anti-CTGF antibodies to select those that bound to CTGF with reasonable affinity and exhibited activity in both *in vitro *assays and *in vivo *models of disease. FG-3019 was chosen as a clinical candidate that has now progressed to phase II testing. FG-3019 binds to the second domain of CTGF of all species tested. We have used FG-3019 in animal models of disease to test whether inhibition of CTGF could reverse pathophysiologic tissue remodeling and fibrosis.

## Discussion

### CTGF is required for the fibrotic activity of TGFβ

**Figure 3:**
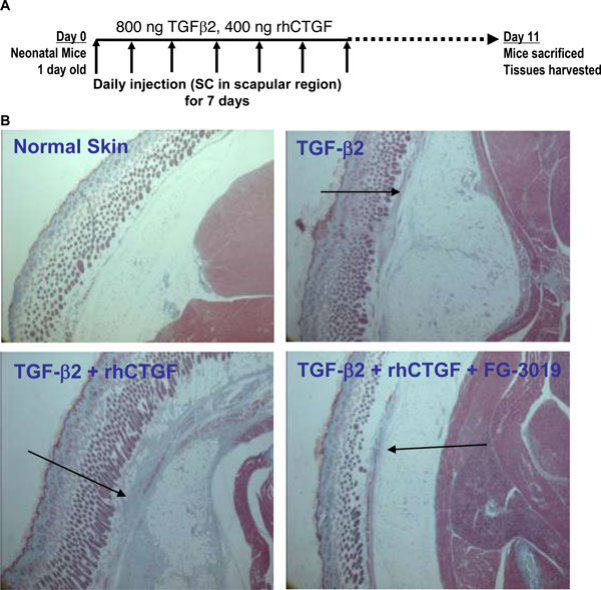
**CTGF is essential for persistent fibrosis**. One day old neonatal mice were injected SC daily for 7 days with 800 ng TGFβ2 alone or with 400 ng CTGF. FG-3019 was also administered to one group that received CTGF. The injections were then stopped for 4 days and the mice were sacrificed to examine the deposition of ECM components and cellular invasion. Panel A: the experimental design. Panel B: A cross section of the skin, SC space and underlying muscle from representative mice is shown. The arrows point out fibrotic response to the various treatments.

In 1999, Mori *et al*. reported that CTGF was required to observe persistent fibrosis after injection of TGFβ into rodents [33]. We and others have been able to confirm their observations in several different experimental models, including one that closely resembles the experiment they performed (Figure 3). One day old neonatal mice were injected subcutaneously in the scapular region daily for 7 days with TGFβ2 or TGFβ2 plus CTGF. The mice were then maintained for another 4 days until they were sacrificed and examined for deposition of ECM and cellular infiltration. As pointed out by the arrows in Figure 3 (panel B), TGFβ induced a modest fibrotic response while that induced by the combination of TGFβ plus CTGF was obviously greater. Co-injection of FG-3019 with the TGFβ and CTGF suppressed all of the response to the presence of CTGF, demonstrating that the antibody was capable of completely inhibiting the biological activity of CTGF.

**Figure 4:**
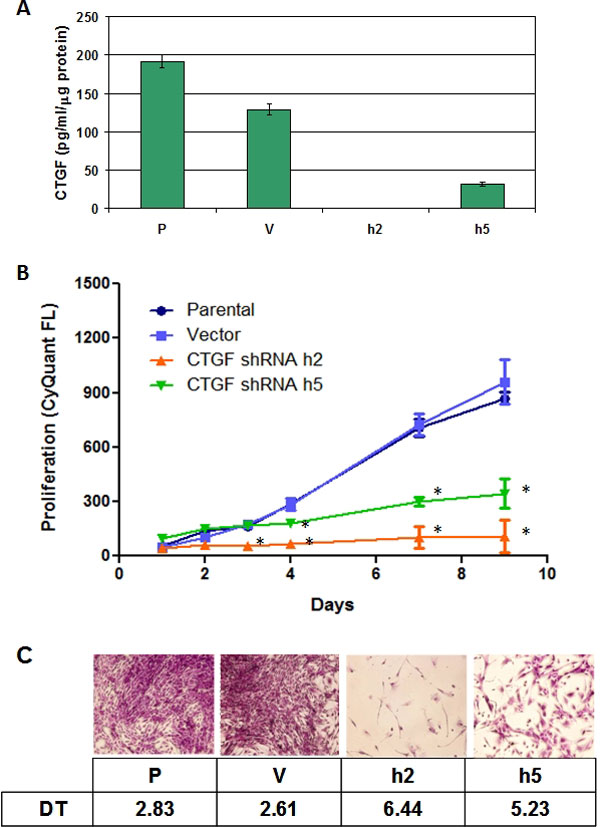
**CTGF knock-down with shRNA inhibits serum-dependent cell proliferation of cardiac fibroblasts**. Primary cardiac fibroblasts (P) were infected with lentiviruses encoding 2 different CTGF shRNAs (h2 and h5 from OpenBiosystems) or with a lentivirus generated from an empty vector (V) as control. Panel A: CTGF protein expression was measured by ELISA in culture supernatants. Panel B: 96 hrs after infection, cells were re-plated in quadruplicate wells of a 96-well plate in medium containing serum and defined growth supplements and incubated for up to 10 days. Cell proliferation was measured by Cyquant Fluorescense Intensity at the indicated timepoints. Panel C: Parallel cultures of cells were stained with crystal violet at day 10 and doubling time (DT) was determined by linear regression analysis on the data shown in B. * p < 0.001 *vs*. vector by ANOVA.

The mechanism by which CTGF enables persistent fibrosis upon stimulation by TGFβ has never been elucidated, due at least in part to the very complex mechanism of action (MOA) of CTGF. In order to address one aspect of the possible MOA, the role of CTGF in fibroblast proliferation was explored. Primary human cardiac fibroblasts (ScienCell Research Laboratories) were examined for serum-dependent production of CTGF and shown to produce measurable amounts (Figure 4). The amount of CTGF produced in these cells could be decreased by infecting them with lentiviruses expressing CTGF shRNAs (Open Biosystems). Knock down of CTGF expression in these primary human cardiac fibroblasts suppressed their ability to grow in medium containing serum and added supplements, and after 9 days in culture, the number of cells correlated to the amount of CTGF produced (Figure 4, Panel B). Assessment of the doubling time of these cells showed that cells with lower CTGF expression levels proliferated more slowly than cells with normal expression levels, and the doubling time appeared to correlate to the amount of CTGF expressed (Figure 4, Panel C). These data indicate that autocrine CTGF expression is critical for the ability of these fibroblasts to proliferate in response to serum and growth supplements.

**Figure 5:**
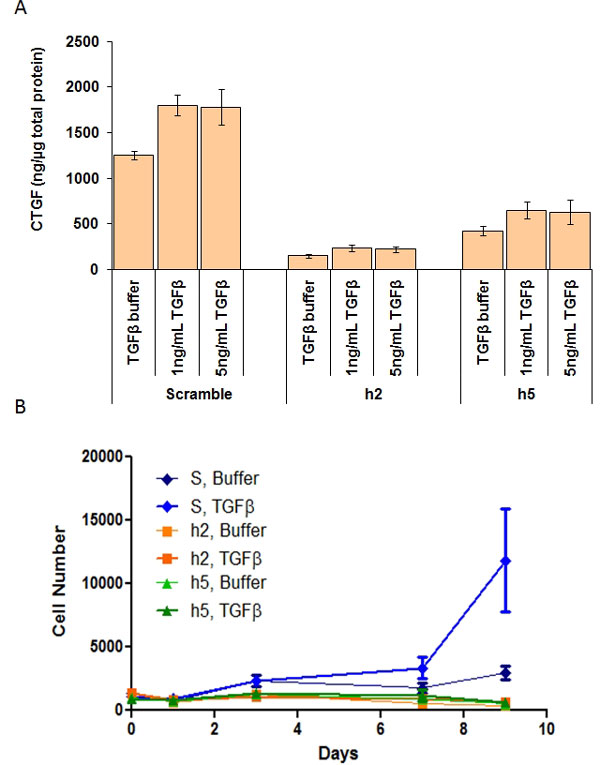
**CTGF knock-down with shRNA inhibits TGFb-dependent cell proliferation of cardiac fibroblasts**. Primary cardiac fibroblasts (P) were infected with lentiviruses encoding CTGF shRNAs (h2 or h5) or with a lentivirus expressing a scrambled shRNA as control. Cells were serum starved for 24 hrs and replated in medium containing growth supplements and the indicated concentrations of TGFb (TGFb). Panel A: CTGF protein expression was measured after 7 days. Panel B: Cell proliferation was measured by Cyquant at the indicated timepoints.

To evaluate the importance of CTGF for TGFβ-induced proliferation of these fibroblasts, cells were infected with lentiviruses expressing CTGF shRNAs or a scrambled negative control shRNA (Figure 5). In serum-starved cells infected with the scrambled shRNA, TGFβ induced expression of more CTGF than in unstimulated fibroblasts. The CTGF shRNA suppressed CTGF expression in cells in control medium or medium supplemented with TGFβ (Figure 5, Panel A). TGFβ stimulation of fibroblasts infected with the lentivirus expressing the scrambled shRNA strongly increased the number of cells after 7 days in culture. In contrast, TGFβ stimulation of fibroblasts infected with the lentiviruses expressing the CTGF shRNAs exhibited no response to TGFβ, and the number of cells after 7 days in culture was lower than the number of cells in unstimulated fibroblasts containing the scrambled shRNA (Figure 5, Panel B). These data suggest that CTGF expression is essential for fibroblast proliferation, and in the absence of CTGF, fibroblasts are unresponsive to stimuli like TGFβ that would normally strongly enhance their proliferation.

### Inhibition of CTGF can reverse fibrosis

Data from experiments in several organ systems suggest that inhibition of CTGF can not only prevent, but can also inhibit fibrosis. For example, in liver fibrosis models CTGF is known to increase in response to toxins such as CCl_4 _[34] or dimethyl nitrosamine [35] or upon bile duct ligation [34]. Inhibition of CTGF expression in a rodent model of CCl_4_-induced liver fibrosis with siRNA can prevent development of fibrosis [36]. If the CTGF siRNA is administered to mice after significant increases of α-smooth muscle actin (αSMA, a marker of myofibroblasts) abundance and collagen deposition can be detected in their liver, the fibrosis appears to regress within two weeks [37].

To examine the cardiovascular complications of diabetes, rats were made diabetic with a single dose of streptozotocin. After 6 weeks of diabetes, the rats were divided into groups and treated for 6 weeks with FG-3019 (10 mg/kg tiw) or the angiotensin converting enzyme inhibitor captopril (75 mg/kg qd). The function of the hearts of the rats in the various groups was then measured prior to their sacrifice and removal of their carotid arteries for measurement of vascular stiffness. After 6 weeks of diabetes, the stiffness of the carotid arteries in the axial orientation was greater than that of healthy control animals. The axial stiffness continued to increase during the next 6 weeks of diabetes. In rats that began receiving captopril after 6 weeks of diabetes, further progression of vascular stiffening was halted, and the axial stiffness remained unchanged from that observed at 6 weeks. In contrast, in rats that received FG-3019 for 6 weeks beginning 6 weeks after they became diabetic, the axial stiffness of the carotid arteries in these diabetic rats reverted to that of healthy control animals. In addition the function of the heart could also be shown to be normalized in animals that received FG-3019, but not in those administered captopril [38]. These data indicate that cardiovascular remodeling associated with the complications of diabetes can be reversed upon treatment with FG-3019.

To evaluate the effect of FG-3019 in pulmonary fibrosis, a radiation-induced model was used and preliminary data were reported at an American Thoracic Society meeting [39-41]. Pulmonary fibrosis was initiated with a single, full thorax irradiation (20 Gy) to mice and FG-3019 was administered for 8 weeks beginning at various times before or after irradiation. Lung density of all surviving mice was monitored by computed tomography (CT).

Irradiation induced lung remodeling beginning around 12 weeks, and FG-3019 attenuated this remodeling in a schedule-dependent manner. Lung density in the group administered FG-3019 beginning 16 weeks after irradiation had already significantly increased at the time that FG-3019 administration began, and decreased with treatment, indicating reversal of the radiation-induced changes [39].

Thus, inhibition of CTGF in liver, the cardiovascular system or the lungs has the potential to reverse tissue remodeling and the process of fibrosis. Together, the results from these studies suggest that inhibition of CTGF may benefit any disease in which tissue remodeling is important. For example, pancreatic cancers are known to be very desmoplastic [42] and to exhibit strong CTGF expression [43,44]. Data suggest that CTGF can have both direct and indirect effects on pancreatic cancers [45-48]. Therefore, the use of FG-3019 for treatment of pancreatic cancer is being explored in parallel with hepatitis B-induced liver fibrosis and idiopathic pulmonary fibrosis.

## Competing interests

The authors are all current employees of FibroGen, Inc., but have no other competing interests.
